# Effects of cyclic stretch on the molecular regulation of myocardin in rat aortic vascular smooth muscle cells

**DOI:** 10.1186/1423-0127-20-50

**Published:** 2013-07-15

**Authors:** Chiung-Zuan Chiu, Bao-Wei Wang, Kou-Gi Shyu

**Affiliations:** 1 School of Medicine, Fu-Jen Catholic University, New Taipei, Taiwan; 2 Division of Cardiology, Shin Kong Wu Ho-Su Memorial Hospital, 95 Wen-Chang Rd, Taipei, Taiwan; 3 Graduate Institute of Clinical Medicine, College of Medicine, Taipei Medical University, Taipei, Taiwan

**Keywords:** Myocardin, Stretch, Vascular smooth muscle cells, ERK pathway

## Abstract

**Background:**

The expression of myocardin, a cardiac-restricted gene, increases during environmental stress. How mechanical stretch affects the regulation of myocardin in vascular smooth muscle cells (VSMCs) is not fully understood. We identify the mechanisms and pathways through which mechanical stretch induces myocardin expression in VSMCs.

****Results**:**

Rat VSMCs grown on a flexible membrane base were stretched to 20% of maximum elongation, at 60 cycles per min. An *in vivo* model of aorta-caval shunt in adult rats was also used to investigate myocardin expression. Cyclic stretch significantly increased myocardin and angiotensin II (AngII) expression after 18 and 6 h of stretch. Addition of extracellular signal-regulated kinases (ERK) pathway inhibitor (PD98059), ERK small interfering RNA (siRNA), and AngII receptor blocker (ARB; losartan) before stretch inhibited the expression of myocardin protein. Gel shift assay showed that myocardin-DNA binding activity increased after stretch. PD98059, ERK siRNA and ARB abolished the binding activity induced by stretch. Stretch increased while myocardin-mutant plasmid, PD98059, and ARB abolished the promoter activity. Protein synthesis by measuring [^3^H]proline incorporation into the cells increased after cyclic stretch, which represented hypertrophic change of VSMCs. An *in vivo* model of aorta-caval shunt also demonstrated increased myocardin protein expression in the aorta. Confocal microscopy showed increased VSMC size 24 h after cyclic stretch and VSMC hypertrophy after creation of aorta-caval shunt for 3 days.

**Conclusions:**

Cyclic stretch enhanced myocardin expression mediated by AngII through the ERK pathway in cultured rat VSMCs. These findings suggest that myocardin plays a role in stretch-induced VSMC hypertrophy.

## Background

In recent years, vascular smooth muscle cell (VSMC) hypertrophy has been increasingly associated with the development of atherosclerotic disease [1]. Hypertrophy of VSMCs may result in plaque rupture and vulnerability in atherosclerosis [2]. Hypertrophy of VSMCs may be induced by certain cardiac-restricted genes through specific pathways under different types of environmental stress [3-5].

Myocardin is a potent cardiovascular regulated gene and transcriptional cofactor, which functionally synergizes with serum response factor (SRF; a transcriptional factor) and has been documented to have measurable effects on cardiac embryo development and both VSMC and cardiomyocyte hypertrophy [6-10]. An earlier study showed myocardin knockout mice resulted in embryonic lethality at E10.5, and was associated with failed VSMC differentiation [7]. Previous studies performed on rat carotid artery VSMCs following vascular injury have demonstrated that myocardin can selectively regulate SRF binding to the degenerate CArGs on VSMC actin to increase transcriptional activity. Loss of myocardin may contribute to the supression of actin expression in response to vascular injury. Vascular injury has been associated with induced expression of myocardin [11]. In addition, transfection with dominant-negative forms and small interfering RNA (siRNA) of myocardin in cultured VSMC decreased transcription of VSMC maker genes. Previous reports also indicated that either exogenous addition or stress-induced expression of angiotensin II (AngII) secretions would result in myocardin expression and subsequent VSMC or cardiac myocyte hypertrophy [12-15]. So myocardin itself may act as a cardiac regulated gene and cooperate with AngII in regulating VSMC or/and cardiac myocyte hypertrophy through specific signal transduction pathways under conditions of stress or injury to the cardiovascular system.

The application of cyclic stretch to cultured VSMCs has been widely used as an *in vitro* experiment to study molecular events in response to mechanical overload [16-20]. It has previously been reported that cyclic mechanical stretch induced hypertrophy in VSMCs [21-24]. Cells in the cardiovascular system are permanently subjected to mechanical forces due to the pulsatile variation of blood flow and shear force, created by the beating heart. These hemodynamic forces play an important role in the regulation of vascular development, remodeling, repair and formation of atherosclerotic stenosis [25-28]. Mechanical stretch can modulate several different cellular functions in VSMCs. These functions may include cell proliferation and differentiation, migration, survival or apoptosis, vascular remodeling, as well as autocrine or paracrine functions [29,30]. This study aimed to identify the cellular and molecular effects of mechanical stretch on VSMCs regulated by myocardin, and to identify its signal transduction pathway and relationship with AngII. Knowing the impact of mechanical stretch on the cardiovascular system is crucial to the understanding of the pathogenesis of cardiovascular diseases, and a key to providing new insight into the prevention and therapy of cardiovascular diseases.

Previous reports have provided strong evidence that myocardin plays an important role in VSMC hypertrophy related to AngII secretion [12]. However, no previous study has shown how cyclic mechanical stretch affects myocardin in the hypertrophy of VSMCs. Thus, in this study, we firstly investigated the mechanism of myocardin expression in cyclic mechanical stretch. Secondly, we investigated the effect and signal transduction pathway of myocardin expression induced by cyclic stretch.

## Methods

### Vascular smooth muscle cell culture

Primary cultures of VSMC were grown by the explant technique from the thoracic aorta of 200–250 g male Sprague–Dawley rats, as previously described [31,32]. Cells were cultured in medium containing 20% fetal calf serum, 0.1 mmol/L non-essential amino acids, 1 mmol/L sodium pyruvate, 4 mmol/L L-glutamine, 100 U/mL penicillin, and 100 mg/mL streptomycin at 37°C under 5% CO2/95% air in a humidified incubator. When confluent, monolayers of VSMCs were passaged every 6–7 days after trypsinization and were used for experiment from the 4^th^ to 6^th^ passages. These 4^th^ to 6^th^ passage cells were then cultured in Flexcell I flexible membrane dishes in medium containing 0.5% fetal calf serum, and the cells were incubated for a further 2 days to render them quiescent before initiating each experiment. The study was reviewed and approved by the Institutional Animal Care and Use Committee of Shin Kong Wu Ho-Su Memorial Hospital and conforms to Guide for the Care and Use of Laboratory Animals published by the US National Institutes of Health (NIH Publication No. 85–23, revised 2011).

### *In vitro* cyclic stretch on cultured vascular smooth muscle cells

The strain unit Flexcell FX-2000 (Flexcell International Co., NC, USA) consists of a vacuum unit linked to a valve controlled by a computer program. VSMCs cultured on the flexible membrane base were subjected to cyclic stretch produced by this computer-controlled application of sinusoidal negative pressure, as previously characterized and described in detail [33,34]. A 10% or 20% cyclic stretch was performed with a frequency of 1 Hz (60 cycles/min).

### Antibodies and reagents

Rabbit polyclonal antibodies against myocardin, mouse monoclonal antibodies (mAbs) against c-Jun N-terminal kinase (JNK) and anti-GAPDH antibodies were obtained from Santa Cruz Biotechnology (Santa Cruz, CA). Mouse mAbs against p38 mitogen-activated protein kinase (MAPK), extracellular signal-regulated kinase (ERK), and phospho-ERK were purchased from BD Bioscience Pharmingen (San Diego, CA). PD98059, SB203580, and SP600125 were purchased from Calbiochem (San Diego, CA). All other chemicals of reagent grade were obtained from Sigma (St Louis, MO). The roles of JNK, p38 MAPK, and ERK in stretch-induced myocardin expression were determined by pretreatment of the VSMCs with 25 μM SP600125, 3 μM SB203580, or 50 μM PD98059 for 30 min before cyclic stretch. SP600125 is a potent, cell-permeable, selective, and reversible inhibitor of JNK. SB203580 is a highly specific, cell-permeable inhibitor of p38 MAPK. PD98059 is a specific and potent inhibitor of the ERK pathway. The AngII and AngII antibodies came from Bachem AG (Torrance, CA). To examine the effect of ARB (AngII type 1 receptor blocker), VSMCs were treated with 100 nM losartan (Merck & Co, Inc).

### Western blot analysis

Western blot was performed as previously described [35].

### Real-time RT-PCR

Total RNA was isolated from VSMCs using the single-step acid guanidinium hiocyanate/phenol/chloroform extraction method. The cDNA produced by reverse transcription (RT) was used to generate myocardin probes by polymerase chain reaction (PCR) as previously described [17]. Primers were designed for detection of myocardin gene expression. The primers for myocardin were: 5′-GGACTGCTCTGGCAACCCAGTGC-3′; reverse: 5′-CATCTGCTGACTCCGGGTCATTTGC - 3′. GAPDH gene expression was used as internal controls. The primers for GAPDH were: 5′-GAGAGGCTCTCTGTCGACTAC-3′; reverse: 5′-TAGTGTAGGTTGGGCGCTCAA-3′.

### RNA interference

VSMCs were transfected with 800 ng of ERK- and myocardin-annealed siRNA (Dharmacon, Lafayette, CO). ERK siRNAs are target- specific 20- to 25-nt siRNAs designed to knock down gene expression. The siRNA sequences were 5′-GACCGGAUGUUAACCUUUAUU (sense) and 5′-PUAAAGGUUAACA UCCGGUCUU (antisense) for ERK. The myocardin siRNA sequences were 5′-UGCAACUGCAGAAGCAGAAUU (sense) and 5′-UGCAACUGGUCUUGCAGAAUU (antisense). As a negative control, a non-targeting (control) siRNA (Dharmacon) was used. For transfection of rat VSMCs with siRNA oligonucleotides, we used Effectene transfection reagent according to the manufacturer’s instructions (Qiagen, Valencia, CA). After incubation at 37°C, cells were subjected to stretch and analyzed by Western blot.

### Measurement of AngII concentration by enzyme-linked immunosorbent assay (ELISA)

Conditioned medium from VSMCs subjected to cyclic stretch and those from unstretched cells were collected for AngII measurement. The level of AngII was measured by a quantitative sandwich enzyme immunoassay technique (R&D Systems, Minneapolis, MN, USA). The lowest limit of AngII ELISA kit was 52 pg/mL.

### Electrophoretic mobility shift assay (EMSA)

Nuclear protein concentrations from cultured VSMCs were determined by the Bradford method as commercialized by Santa Cruz Biotechnology and EMSA was performed as previously described [13]. Consensus and control oligonucleotides were labeled by polynucleotide kinase incorporation of [γ-^32^P] ATP. In each case, mutant or cold oligonucleotide was used as a control to compete with the labeled sequences. The oligonucleotide sequence of consensus binding site for SRF was sense: 5′- GGA TGT CCA TAT TAG GAC ATC T-3′ and reverse: 5′-CCT ACA GGT ATA ATC CTG TAG A-3′. The mutant oligonucleotide sequence was 5′- GGA TGT CCA TAT TAT TAC ATC T-3′.

### Promoter activity assay

A −968 to +44 bp rat myocardin promoter construct was generated as previously described [13]. The myocardin promoter contains myocardin binding sites for SRF (sequences: CGGTTTAGGG) located at −514 to −505 bp of the promoter region. We used the binding sites to detect the transcriptional activity of myocardin. For construction of the mutant SRF binding region of myocardin, we changed the sequences located at −507 to −506 bp from CGGTTTAGGG to CGGTTTATTG by using a mutagenesis kit (Stratagene, La Jolla, CA). Site-specific mutations were confirmed by DNA sequencing. Plasmids were transfected into VSMCs using a low-pressure accelerated gene gun (Bioware, Taipei, Taiwan). Rat genomic DNA was amplified with forward (GGACTGCTCTGGCAAC CCAGT GC) and reverse (CATCTGCTGACTCCGGG TCATTTGC) primers. The amplified product was digested with *Mlu* I and *Bgl* II restriction enzymes and ligated into pGL3-basic luciferase plasmid vector (Promega, Madison, WI) digested with the same enzymes. In brief, 2 μg of plasmid DNA was suspended in 5 mL of PBS and was delivered to the cultured VSMCs at a helium pressure of 15 psi. The transfection efficiency using this method is 30%. Following 12 h of cyclic stretching, cell extracts were prepared using the Dual-Luciferase Reporter Assay System (Promega) and measured for dual luciferase activity by luminometer (Turner Designs, Sunnyvale, CA, USA).

### Migration assays

The migration activity of VSMCs was determined using the growth-factor-reduced Matrigel invasion system (Becton Dickinson), following the protocol provided by the manufacturer. The migration assay was performed as previously described [17].

### Determination of protein synthesis

Protein synthesis was examined by measuring [^3^H]proline incorporation into the cells. Cultured VSMCs were divided into the following groups: (1) control group: the cells were cultured in serum-free DMEM; and (2) mechanical stretch group (20% cyclic stretch) added to serum-free medium. Each experiment was repeated 6 times. VSMCs were first grown in DMEM with 10% FBS and 200 mg/L L-glutamine, and then seeded in 24-well plates at 1 × 10^5^ cells/well in DMEM + 10% FBS. After synchronization of VSMCs, the medium was changed to DMEM without serum. VSMCs were treated with cyclic stretch and exposed to [^3^H]proline at the concentration of 1 μCi/well for the last 12 h of the 24 h incubation period. After the incubation, the cells were washed with ice-cold PBS and 10% trichloroacetic acid. Acid-insoluble [^3^H]proline was collected on glass fiber filters (Whatman, Kent, UK) and determined by a liquid scintillation counter (LS 6500, Beckman, Fullerton, CA , USA).

### Rat model of aorta-caval shunt

The aorta-caval shunt was produced as previously described [17]. The vena cava and aorta were exposed via abdominal midline incision. In brief, the aorta was punctured at the union of the segment two-thirds caudal to the left renal artery and one-third cephalic to the aortic bifurcation, with an 18-gauge disposable needle held with a plastic syringe. The needle was advanced into the aorta, perforating its adjacent wall and penetrating the vena cava. The induced aorta-caval shunt produced a ratio of 1.7 of pulmonary to systemic flow. Sham-operated control animals were prepared in a similar manner, except that the aorta was not punctured.

### Statistical analysis

All results were expressed as means ± SEM. Statistical significance was evaluated using variance (GraphPad Software Inc., San Diego, CA, USA). Dunnett’s test was used to compare multiple groups to a single control group. Tukey-Kramer comparison was used for pairwise comparisons between multiple groups after ANOVA. A value of P < 0.05 was considered to denote statistical significance.

## Results

### Cyclic stretch enhances myocardin protein and mRNA expression in vascular smooth muscle cells

**Figure 1:**
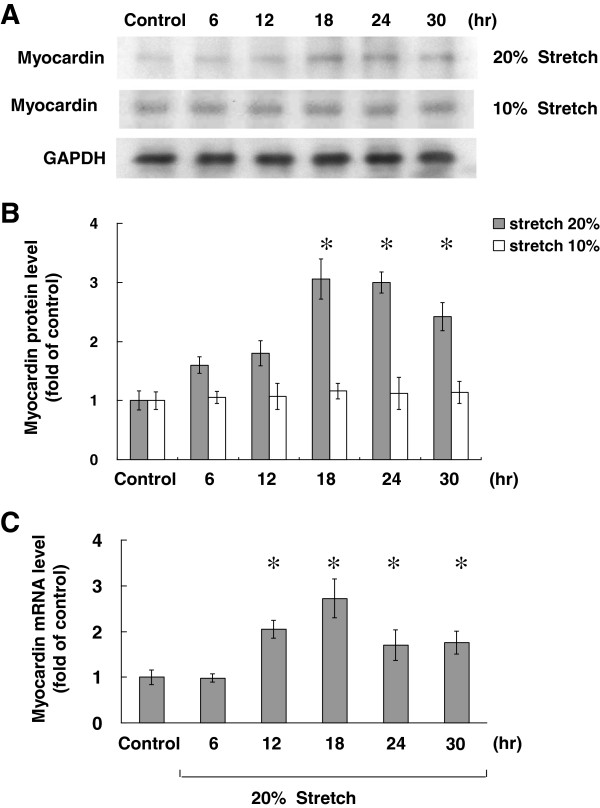
**Cyclic stretch induces myocardin protein and mRNA expressions in VSMCs.** Western blots for myocardin in VSMCs subjected to cyclic stretch by 10% or 20% **(A)** for varying periods of time. VSMCs were kept as control or subjected to cyclic stretch and their protein **(B)** and mRNA **(C)** expressions were determinated by western blot and quantitative real time PCR analyses, respectively. Data are shown as mean ± SEM from 5 independent experiments. *P < 0.01 vs. control.

The level of myocardin protein began to increase as early as 6 h after stretch to 20% elongation, reaching a maximum of 3.1-fold over the control by 18 h, remaining elevated up to 24 h and tending to decline at 30 h. When VSMCs were stretched at 10% elongation, the level of myocardin protein was similar to that of controls without stretch (Figure 1A and B). The real-time PCR showed that myocardin mRNA expression increased maximally after 18 h of stretch at 20% elongation (Figure 1C). These results indicated that cyclic stretch induced myocardin expression in VSMCs.

### Stretch-induced myocardin protein expression in vascular smooth muscle cells is mediated by the ERK pathway

**Figure 2:**
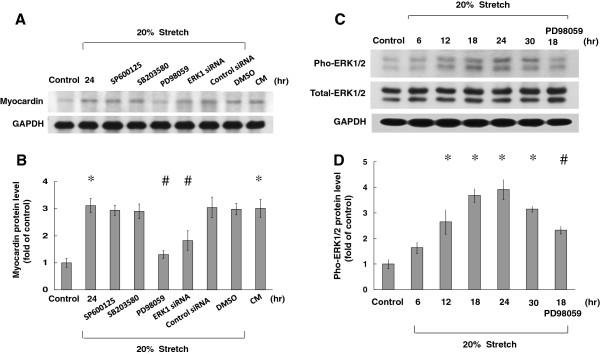
**Cyclic stretch-induced myocardin expression is mediated by the ERK pathway.** (**A** and **B**) ERK pathway inhibitor (PD98059) and ERK siRNA knocked down total ERK protein expression. (**A**) Western blots for myocardin protein in VSMCs subjected to cyclic stretch in the absence or presence of inhibitors, siRNA, and vehicle (DMSO; Dimethyl sulfoxide 0.1%). CM = conditioned medium. (**B**) Quantitative analysis of myocardin protein levels. The values from stretched VSMCs have been normalized to values in control cells (n = 5 per group). *P < 0.01 vs. control. (**C** and **D**) Phosphorylation of ERK mediated stretch-induced myocardin expression, which was blocked by PD98059. Rat VSMCs were subjected to stretch for different periods of time in the presence or absence of inhibitors, and cell lysates were collected for western blot analysis using antibodies for total and phospho-ERK. T-ERK = total ERK. P-ERK = ERK phosphorylation. *P < 0.01 *vs*. control. ^#^*P* < 0.01 *vs*. 18 h; (n = 3).

To identify the possible signal pathway mediating the stretch-induced myocardin expression in VSMCs, the VSMCs were stretched 20% for 24 h in the presence and absence of inhibitors or siRNA. As shown in Figure 2, the stretch-induced increases of myocardin proteins were significantly blocked when PD98059 (ERK pathway inhibitor; 50 μM) and ERK siRNA were added 30 min before stretch. The myocardin proteins induced by stretch were not affected by the addition of SP600125 (20 μM) or SB203580 (30 μM). To test the specific effect of ERK MAPK (Mitogen-Activated Protein Kinase) pathway on mediating the expression of myocardin, ERK siRNA was transfected to VSMCs before cyclic stretch. Moreover, ERK siRNA also completely blocked the myocardin expression induced by stretch (Figure 2B). The DMSO (Dimethyl sulfoxide) alone as a vehicle control and control siRNA did not affect the myocardin expression induced by cyclic stretch. These findings implied that the ERK pathway, but not JNK or p42/p44 MAP kinases, mediated the induction of myocardin proteins by stretch in VSMCs. The conditioned medium from stretched VSMCs could induce the same increase in myocardin protein expression in non-stretched VSMCs (Figure 2A and B). These findings suggest that cyclic stretch regulated myocardin protein expression in VSMCs possibly via autocrine or paracrine mechanisms.

### Cyclic stretch increases the phosphorylation of ERK protein in rat vascular smooth muscle cells

We also found that ERK protein phosphorylation increased to its maximal level 24 h after 20% cyclic stretch and declined gradually. The ERK pathway inhibitor (PD98059) could effectively block the phosphorylation of ERK protein (Figure 2C and D).

### Cyclic stretch stimulates secretion of angiotensin II from vascular smooth muscle cells

**Figure 3:**
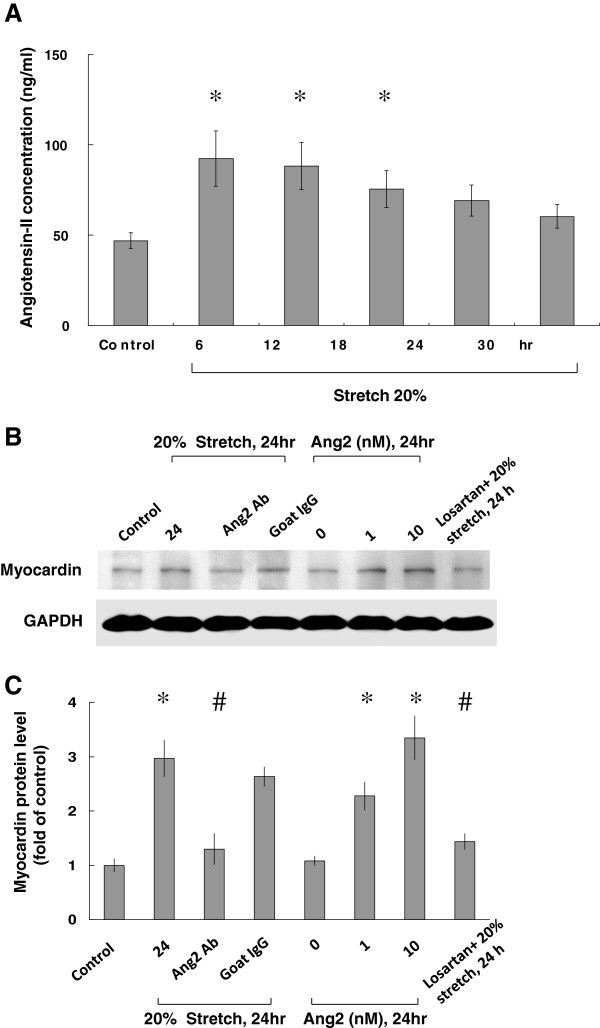
**AngII mediates stretch-induced myocardin expression in rat VSMCs.** (**A**) AngII was measured in the culture medium by a quantitative, competitive ELISA, using a specific anti-AngII antibody. AngII levels increased significantly 6 h after 20% stretch. (**B** and **C**) Either 20% stretch or exogenous addition of AngII increased myocardin expression, and was inhibited by AngII antibodies or ARB (losartan). In this experiment, Western blots for myocardin protein in VSMCs subjected to cyclic stretch or exogenous addition of AngII in the absence or presence of inhibitors (AngII Ab or losartan) were performed. GAPDH was shown for equal amounts of protein loading in each lane. *P < 0.01 *vs*. control. ^#^*P* < 0.01 *vs*. 24 h after stretch or 10 h after exogenous addition of AngII (n = 3).

As shown in Figure 3A, cyclic stretch significantly began to increase the AngII secretion from VSMCs and reached a peak at 6 h after stretch. Later, AngII levels declined gradually until 30 h (Figure 3A). Myocardin protein expression induced by cyclic stretch could effectively suppressed by AngII antibodies (Figure 3B and C). These results indicated that stretch causes VSMCs to secrete AngII.

### Exogenous addition of angiotensin II increases myocardin protein expression

To investigate the direct effect of AngII on myocardin expression in VSMCs, AngII at different concentrations was administered to the cultured medium for 24 h. As shown in Figure 3B and C, the effect of AngII on myocardin protein expression was dose-dependent. These findings suggested that exogenous addition of AngII also enhances myocardin expression without cyclic stretch. Addition of losartan 30 min before stretch significantly blocked the expression of myocardin induced by cyclic stretch for 24 h.

### Cyclic stretch increases myocardin binding activity

**Figure 4:**
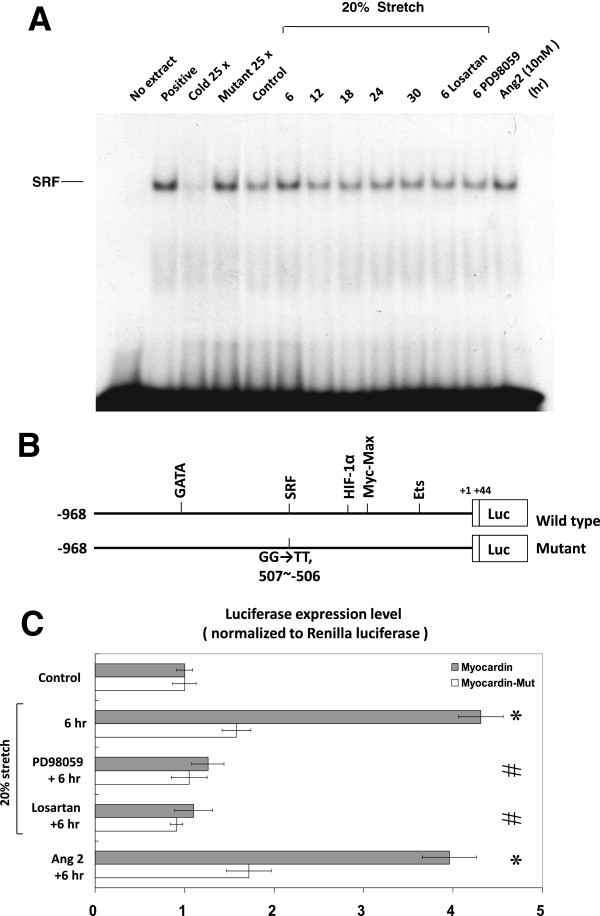
**Effects of cyclic stretch on myocardin binding activity and promoter activity in VSMCs.** Binding activity between myocardin and SRF and genetic transcription activity at SRF binding site of myocardin promoter increase in VSMCs under 20% cyclic stretch. (**A**) EMSA showed increased binding between myocardin and SRF under 20% cyclic stretch, which was suppressed by losartan and PD98059. Exogenous addition of AngII (10 nM) without cyclic stretch also increased the binding between myocardin and SRF. (**B** and **C**) Luciferase reporter assay revealed 20% cyclic stretch increased transcriptional activity at SRF binding sites of myocardin promoter when compared with the myocardin mutant, which was suppressed by PD98059 and losartan. Exogenous addition of AngII (10 nM) without cyclic stretch also increased the transcriptional activity in VSMCs. *P < 0.01 *vs*.control. ^#^*P* < 0.01 *vs*. 6 hr (n = 3 per group).

Cyclic stretch of VSMCs for 6–18 h significantly increased the DNA-protein binding activity of myocardin to SRF (Figure 4A). An excess of unlabelled myocardin oligonucleotide competed with the probe for binding myocardin protein, whereas an oligonucleotide containing a 2 bp substitution in the myocardin binding site did not compete for binding. Addition of ARB (losartan) and ERK pathway inhibitor (PD98059) 30 min before stretch abolished the DNA-protein binding activity induced by cyclic stretch. Exogenous administration of AngII to the VSMCs without stretch also increased myocardin-DNA binding activity (Figure 4A). These results demonstrated that stretch enhanced myocardin binding activity in VSMCs.

### Cyclic stretch increases myocardin promoter activity through the ERK pathway

To study whether the myocardin expression induced by stretch is regulated at the transcriptional level, we cloned the promoter region of rat myocardin (−968 to +44) and constructed a luciferase reporter plasmid (pGL3-Luc). The myocardin promoter construct contains myocardin binding sites. As shown in Figure 4B and C, transient transfection experiments on VSMCs using this reporter gene revealed that stretch for 6 h significantly induced myocardin promoter activity. This result indicated that myocardin expression in VSMCs is induced at transcriptional level during cyclic stretch. When the myocardin binding sites were mutated, the increased promoter activity induced by stretch was abolished. Moreover, addition of ERK pathway inhibitor (PD98059) and ARB (losartan) caused an inhibition of transcription. These results suggested that the binding site in the myocardin promoter is essential for transcriptional regulation by cyclic stretch. In addition, we also found that exogenous addition of AngII without stretch increases the transcriptional activity in VSMCs (Figure 4C).

### Myocardin increases the migration of VSMCs

**Figure 5:**
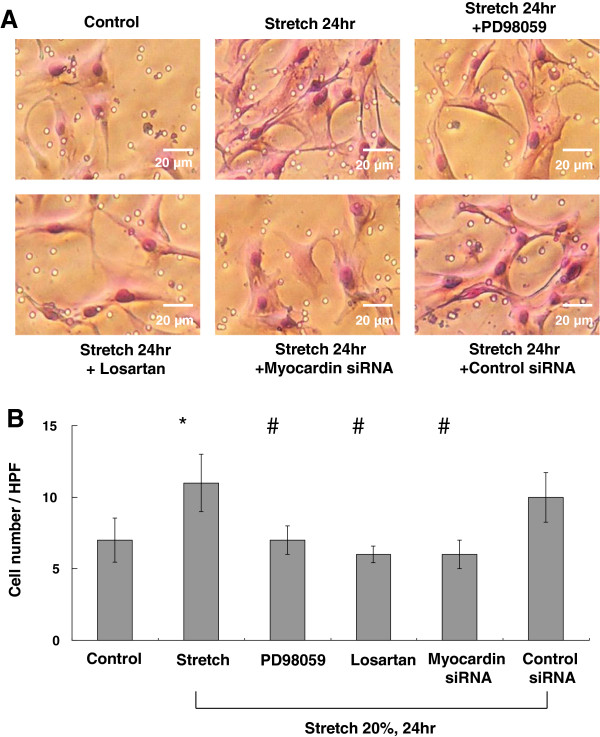
**Effect of stretch on the migration of myocardin in VSMCs.** (**A**) Condition medium (CM) was obtained from VSMCs after cyclic stretch for 24 h. VSMCs migrating through the filter were stained. (**B**) The stained VSMCs were counted in four fields under a 400 X high-power field (HPF). *P < 0.01 *vs*. control. ^#^*P* < 0.05 *vs*. 24 h (n = 3).

Rat VSMCs cultured in the conditioned medium generated from stretched cells migrated significantly through the filter membrane compared with those cultured in non-conditioned medium (Figure 5A and B). Inhibition of myocardin activity was observed by ERK pathway inhibitor (PD98059), ARB (losartan), and myocardin siRNA, which decreased VSMC migration activity (Figure 5A and B). The migration activity of VSMCs was similar in controls and scrambled siRNA-treated groups. These findings suggest that myocardin mediates the migration of VSMCs induced by cyclic stretch.

### Cyclic stretch induces protein synthesis in VSMCs and VSMC hypertrophy

**Figure 6:**
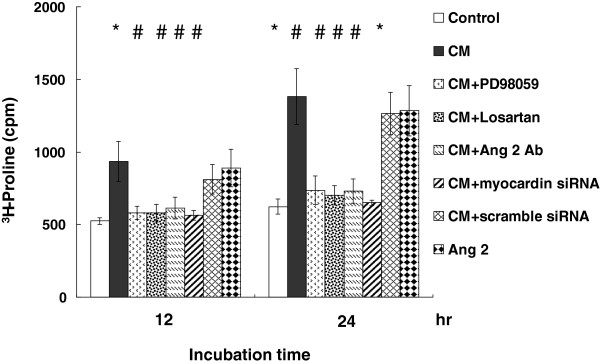
**Protein synthesis in VSMCs increases after cyclic stretch.** Incorporation of ^3^H-proline into VSMCs in condition medium (CM) increased after 20% cyclic stretch and exogenous addition of AngII for 12 to 24 hrs and was suppressed by PD98059, losartan, AngII antibody, and myocardin siRNA. *P < 0.05 *vs*. control. ^#^*P* < 0.05 *vs*. cyclic stretch (n = 6 per group).

**Figure 7:**
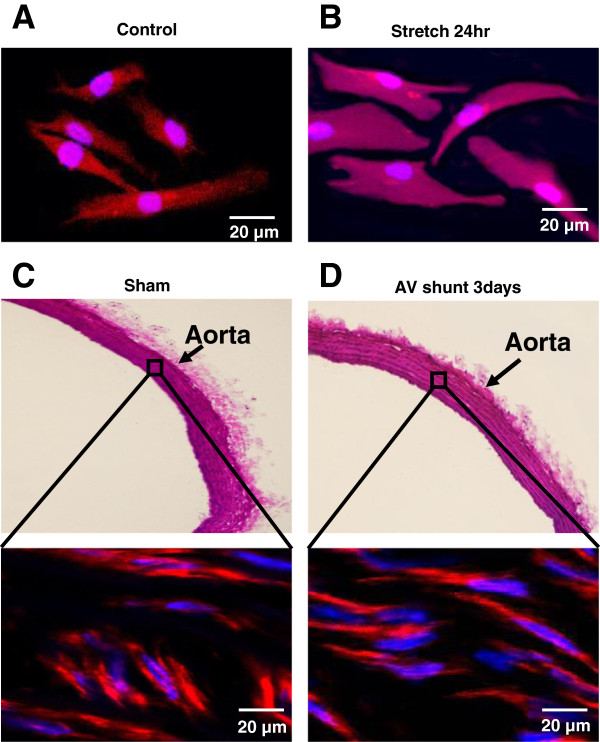
**Cyclic stretch and aorta-caval shunt induce VSMC hypertrophy.** Confocal microscopy showed increased VSMC size 24 hr after cyclic stretch, which represented a hypertrophic change of VSMCs (Figure 7**B**). In addition, VSMC hypertrophy was also noted after creation of aorta-caval shunt for 3 days (Figure 7**D**).

We evaluated protein synthesis in VSMCs by measuring [^3^H]proline incorporation into the cells. Result showed increased protein synthesis in conditioned medium (CM) after cyclic stretch for 12 to 24 hr, which represented hypertrophic change of VSMCs (Figure 6). Pretreatment with ERK pathway inhibitor (PD98059), losartan, AngII antibody, and myocardin siRNA inhibited the protein synthesis induced by cyclic stretch. Exogenous addition of AngII also increased proline incorporation similar to the effect of cyclic stretch. We also used confocal microscopy to identify the effect of VSMC hypertrophy under cyclic stretch (Figure 7A and B). Confocal microscopy showed increased VSMC size 24 hr after cyclic stretch, which represented a hypertrophic change of VSMCs (Figure 7B).

### *In vivo* aorta-caval shunt increases aortic myocardin protein expression

**Figure 8:**
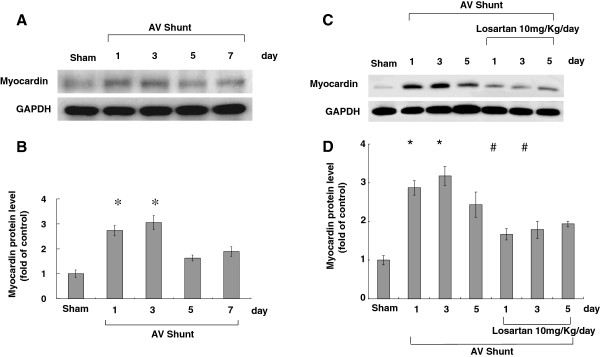
**Effect of in vivo model of aorta-caval shunt (AV shunt) on aortic myocardin protein levels.** (**A**) Western blots for myocardin in rat aorta after short-term induction of AV shunt. (**B**) Quantitative analysis of myocardin protein levels. The values have been normalized to GAPDH measurement and then expressed as a ratio of normalized values to myocardin protein in sham. (**C** and **D**) Treatment with losartan (10 mg/kg/day) throughout day 1 to day 5 after performing aorta-caval shunt signigicantly suppressed myocardin protein expression from day 1 to day 3. *P < 0.01 *vs*. control. ^#^*P* < 0.05 *vs*. 24 h (n = 5).

Aorta-caval shunt was performed to explore whether myocardin expression increased under volume-overload *in vivo*. As shown in Figure 8, the myocardin protein expression in rat aorta significantly increased at days 1 to 3 after performing of aorta-caval shunt. It reached a maximum of 3.0-fold over the sham-operated rat and remained elevated up to 7 days. In addition, treatment with losartan (10 mg/kg/day; from day 1 to day 5) after performing aorta-caval shunt signigicantly suppressed myocardin protein expression (Figure 8C and D). Confocal microcopy also revealed VSMC hypertrophy after creation of aorta-caval shunt for 3 days (Figure 7D).

## Discussion

In this study, we demonstrated several significant or novel findings. Firstly, cyclic stretch upregulates myocardin expression in rat VSMCs; secondly, cyclic stretch induces AngII expression in VSMCs; thirdly, AngII acts as an autocrine factor to mediate the increased myocardin expression induced by cyclic stretch; fourthly, ERK MAP kinase and SRF transcriptional factor are involved in the signaling pathway of myocardin induction; and fifthly, *in vivo* acute hemodynamic overload increases aortic myocardin expression. Myocardin was upregulated in both a time- and load- dependent manner by cyclic stretch. Cyclic stretch of VSMCs increased both myocardin protein and mRNA expression.

In our study, exogenous addition of AngII to non-stretched VSMCs was also sufficient to induce similar myocardin protein expression as that observed in stretched VSMCs. These results provide the first evidence that AngII mediates cyclic stretch-induced expression of myocardin in VSMCs. Our study revealed that AngII acts as an autocrine mediator in response to cyclic stretch in VSMCs. Previously, another study identified that AngII enhanced myocardin expression through AngII type 1 (AT1) receptor results in VSMC hypertrophy [12]. We also have previously demonstrated that hypoxia in cardiomyocytes increased AngII secretion and myocardin expression and finally resulted in cardiac myocyte hypertrophy through the ERK pathway [13]. In this study, we found that cyclic stretch also enhanced myocardin expression by AngII secretion and the ERK pathway, which had not been identified by previous studies.

However, one study found no increased concentration of AngII in the medium collected from porcine VSMCs at 24 and 48 h after 25% stretch [36]. Sotoudeh et al. used pulmonary VSMCs, whereas our study used rat aortic VSMCs. Different species, stretch intension, and stretch time may explain the discrepancy. Our results suggest that AngII is responsible for myocardin-DNA binding in VSMCs. In this study, we demonstrated that cyclic stretch stimulation of myocardin-DNA binding activity required at least phosphorylation of ERK since ERK pathway inhibitor (PD98059) and ERK siRNA abolished the myocardin/SRF binding activity. PD98059, a potent and specific inhibitor of ERK MAP kinase, also inhibited the myocardin expression induced by stretch, whereas inhibitors of p42/p44, p38, and c-JUN MAP kinase did not have this inhibitory effect. Thus, ERK MAP kinase is an important intracellular signaling pathway that regulates myocardin expression. We also demonstrated that ERK siRNA significantly inhibited myocardin expression induced by stretch. ARB likewise had an inhibitory effect on the stretch-induced myocardin expression. Since ARB is an AngII inhibitor, and mechanical stretch is known to affect the production of AngII, [37], our findings potentially indicate that AngII has a role in the induction of myocardin by mechanical stretch. In this study, we demonstrated via promoter activity assay that increased transcriptional activity of myocardin promoter by cyclic stretch was SRF dependent. These data imply that the ERK MAP kinase pathway, but not the other MAP kinase pathway, is the major pathway involved in the induction of myocardin by stretch and that it mediates the increased binding activity of myocardin and transcription to VSMCs.

Mechanical stretch can modulate several different cellular functions in VSMCs. These functions include cell alignment and differentiation, migration, survival or apoptosis, vascular remodeling, and autocrine or paracrine functions [37]. However, use of different kinds of VSMCs (venous or arterial) and various species of animals used in different studies (mouse, rat, rabbit, swine and others), have resulted in sometimes controversial findings [37]. Most of these studies used *in vitro* models. However, the cellular functions induced by in vitro mechanical stretch may not accurately represent cellular function *in vivo*[37]. So, more studies are necessary to identify the real effects of mechanical stretch on VSMC functions and the mechanisms by which they occurr. Our study further confirmed the increased aortic myocardin expression in acute hemodynamic overload as that occurring with aorta-caval shunts. It has been previously reported that myocardin protein expression increased in the carotid artery balloon injury model in rats [11], suggesting myocardin may be enhanced during acute hemodynamic overload *in vivo*. The increased myocardin protein expression following acute hemodynamic overload may contribute to the regulation of vascular repair and remodeling, which involves VSMC proliferation [17].

With regard to the clinical application of cyclic stretch on VSMCs, mechanical stretch activates multiple intracellular signaling networks and regulates gene expressions and functional responses in VSMCs. The cellular and molecular effects of mechanical stretch on vascular cells may provide new insights in the pathogenesis of vascular diseases and therapeutic potentials. Mechanical stretch can modulate several different cellular functions in VSMCs, including cell alignment and differentiation, migration, survival or apoptosis, vascular remodeling, and autocrine and paracrine functions. Arterial VSMCs are aligned primarily in the circumferential direction in the media of the artery. Mechanical stretch from pulsatile blood flow is one of the key factors in regulating vascular remodeling. VSMC migration is important in the development of vascular diseases, including atherosclerosis and post-angioplasty restenosis. VSMC migration is found more frequently in curved and bifurcating blood vessels, which are exposed to non-laminar blood flow, than in straight arterial segments exposed to laminar blood flow. In this, we have also demonstrated that mechanical stretch increased the migration of VSMCs. The gene expression induced by mechanical stretch may be relevant to pathological complications in the cardiovascular system, including atherosclerosis, plaque instability and hypertension. The induction of genes by mechanical stretch may play a role in vascular remodeling. Understanding the molecular mechanisms regulating VSMC remodeling, migration, and proliferation under mechanical stretch supports the clinical application of ACEI (angiotensin-converting enzyme inhibitors), ARB, and statin in cardiac protection and in the prevention of vascular diseases. Therefore knowledge of the impact of mechanical stretch on VSMCs is vital in the understanding of the pathogenesis of cardiovascular diseases and is crucial in providing new insights into the prevention and therapy of cardiovascular diseases.

## Conclusion

In summary, our study reports for the first time that cyclic mechanical stretch enhances myocardin expression in cultured rat VSMCs. The stretch-induced myocardin is mediated by AngII through the ERK pathway.

## Abbreviations

VSMCs: Vascular smooth muscle cells; SRF: Serum response factor; siRNA: Small interfering RNA; AngII: Angiotensin II; mAbs: Monoclonal antibodies; JNK: C-Jun N-terminal kinase; MAPK: Mitogen-activated protein kinase; ERK: Extracellular signal-regulated kinase; ARB: AngII type 1 receptor blockers; RT: Reverse transcription; PCR: Polymerase chain reaction; ELISA: Enzyme-linked immunosorbent assay; EMSA: Electrophoretic motility shift assay; DMEM: Dulbecco’s modified Eagle’s/F12 medium; CM: Conditioned medium; AT1: Receptor: AngII type 1 receptor.

## Competing interests

The authors declare that they have no competing interests.

## Authors’ contributions

CZC participated in the design of the study and drafted the paper. BWW made substantial contributions to conception and design, acquisition of data or analysis, and interpretation of data. KGS participated in the design of the study and final approval of the paper prior to submission. All authors read and approved the final manuscript.
